# Supplementary material for: Imaging feature-based clustering of financial time series
Source: PLoS One. 2023 Jul 26;18(7):e0288836. doi: 10.1371/journal.pone.0288836 (PMC10370745; doi:10.1371/journal.pone.0288836)
Supplement: Attachment — Submitted filename: Response to Reviewers.docx [file pone.0288836.s002.docx]

**Response to reviewers,**

**To Reviewer 1,**

**1. Extensive English editing is required throughout the manuscript.**

Response: Thanks for the meticulous work of the review on this manuscript. We check our script again and find some language mistakes. We hope the revised one could be clarity.

**2. The abstract needs the following improvements
2.1 Clarify the research problem: The abstract should clearly state the specific research problem or research gap being addressed, such as investigating the effectiveness of using 2D modeling technology (e.g., sift features and deep learning methods) for clustering tasks in timeseries analysis.**

Response: the specific research problem is rewrite as,

Timeseries representation underpin our ability to understand and predict the change of natural system. Series are often predicated on our choice of highly redundant factors, and in fact, the system is driven by a much smaller set of latent intrinsic keys. It means that a better representation of data makes points in phase space clearly for researchers. Specially, a 2D structure of timeseries could combine the trend and correlation characters of different periods in timeseries together, which provides more clear information for top tasks.

**2.2 Provide context: The abstract should briefly explain the significance of using imaging features in timeseries clustering tasks and highlight the potential advantages compared to other methods.**

Response: We **highlight the potential advantages compared to other methods as**

Specially, a 2D structure of timeseries could combine the trend and correlation characters of different periods in timeseries together, which provides more clear information for top tasks. In this work, the effectiveness of 2D structure of timeseries is investigated in clustering tasks.

**2.3 Summarize key findings: The abstract should provide a concise summary of the main findings of the study, including the effectiveness or accuracy of the RP imaging series in recognizing abnormal fluctuations in financial timeseries, and any notable insights gained from analyzing the CSI300 and S&P500 indexes.**

Response: **A concise summary of the main findings of the study is added as**

By classifying the CSI300 and S&P500 indexes, we found that the RP imaging series are valid in recognizing abnormal fluctuations of financial timeseries, as the silhouette values of clusters are over 0.6 to 1. Compared with segment methods, the 2D models have the lowest instability value of 0.

**3. Line 97-99: This section should have what makes DL so famous, and which type of recent applications it has to justify its utilization in the present work. I suggest adding smotednn: novel model for air pollution forecasting and aqi classification; cdlstm: a novel model for climate change forecasting; analysis of environmental factors using ai and ml methods; deep learning based modeling of groundwater storage change; deep learning-based supervised image classification using uav images for forest areas classification**

Response: We appreciate the work of DL on climate and environment as

DL have excellent performance on the two complex systems of climate and environment. The deep neural network model carry on time series forecasting of environmental variables [20]. Mohd A. Haq optimizes one novel model SMOTEDNN to address air pollution classification [21]. The improved LSTM could forecast all Himalayan states’ temperature and rainfall values [22]. Further, the LSTM Terrestrial Water Storage Change and Ground Water Storage Change [23]. To deal with 2D questions, deep learning could play in the planning and management of forest areas under unmanned aerial vehicles observations [24].

**4. How the authors addressed the limitations of the recurrent plot method including sensitivity to parameter selection and limited capture of long-term dependencies
5. How the authors addressed the limitations of the GASF method including loss of information and dependency on the choice of distance metric.**

Response: In our experiments, we optimized the RP plot as

In 2.1, the Recurrent Plot method include sensitivity to parameter selection and limited capture of long-term dependencies when there is a super-parameter ε. A more spanned model in this work optimized the plot by dumping the constraint function.

$R_{i,j}^{*}=||x_{i}-x_{j}||, i\text{，}j=1,\cdots,N$.

Then, the Rij* reserves more information of timeseries in the 2d images.

Because the GASF method didn’t show a valid result in the task of financial timeseries as it includes loss of information and dependency on the choice of distance metric, we have not discussed in this work. It maybe a good direction for further research.

**6. Section 2.3 Clustering Algorithm: How authors optimize the K for Kmeans and KMedoid.**

Response: We chose the K according to the base work reference from [7] and [10]

As suggested by [7], the projecting dimension of features on each image is 4200, which is valid to describe timeseries and is suitable for computing. According to this rule, we chose dimensions as 4-15 times the average number of features on each image for comparison. [10] reports a number of 3 categories for stocks, and [7] recommends six groups for the CIS300 and the S&P500. Then, an interval of 3-6 groups was set for the four clustering methods.

**7. Section 2.3.3: The SOM information should briefly summarize the key features or steps of the SOM method, such as the two-layer structure, competitive learning, and the adjustment of vectors based on exponential distance. This would provide a clearer understanding of how SOM works and its potential applicability to clustering tasks.**

Response: We explain the procedure of the SOM algorithm as,

**The SOM algorithm**

For *i* in 1:N numbers of samples

Initial two layers:

Hidden layers: including nodes as cluster centers *C_i_*

Input layers: all selected samples *X_i_*

Learning rate: *β.*

Competition process:

Nodes are activated by computing

D*ij*= Min || *X_i_* - *C_i_* ||

Update:

The activated node will be updated as

C*_i_*= C*_i_* +*β*(X_i_* - *C_i_)*

End

**8. # of parameters are required for the models with FLOPS and the computational complexity.**

Response: the GPU parameter is updated as a GPU of NVIDIA RTX3070 with 22663 GFLOPS.

**9. Limitations and the future scope should be added with more clarity.**

Response: we rewrite the conclusion part for this comment as

There are still some limitations in the current method. Because the SIFT features are local points, they can only capture the relation of joint points in pairs. Although they have included long-term information with imaging series, there are more features of image blocks on imaging series that should be considered for segments relation in long term periods.

In the future, we will adopt segment features in images of financial time series to eliminate the localization of SIFT. The deep learning method is another way to improve the long-term features in images. We hope to construct more wide features in a high-dimension subspace that can emerge the intrinsic changes in financial timeseries.

**10. An experiment environment with computational complexity should be added.**

Response: In a structural program frame, we list **computational complexity as**

Table 1 displays all computational complexity in the experiments. Where *n* is the number of samples，*k* is the number of clusters and *t* is the number of iterations.

**Table 1 computational complexity**

| **Procedure** | **Methods** |
| --- | --- |
| Imaging | RP/*O(n^2^)*, GASF*/O(n^2^)*, GADF/*O(n^2^)*, MTF*/O(n^2^)* |
| Projection | VQ/*O (n^2^),* LLC/*O (n^2^)* |
| Clustering | Kmeans */O(nkt), Kmedoids/O(n(n-k)^2^), Spectral O(nkt), SOM/ O(nkt)* |
| Rubustness | *O(nk)* |

**11. Authors need to provide the merits of this study vs. other review studies.**

**12. The inter-comparison or comparison with other studies is missing, please add them.**

Response: reference to the listed works as,

Shi, Y.; Li, B.; Du, G.; Dai, W. Clustering Framework Based on Multi-Scale Analysis of Intraday Financial Time Series. Physica A: Statistical Mechanics and its Applications 2021, 567, 125728.

D’Urso, P.; De Giovanni, L.; Massari, R. Trimmed Fuzzy Clustering of Financial Time Series Based on Dynamic Time Warping. Annals of operations research 2021, 299, 1379–1395.

D’Urso, P.; Cappelli, C.; Di Lallo, D.; Massari, R. Clustering of Financial Time Series. Physica A: Statistical Mechanics and its Applications 2013, 392 (9), 2114–2129.

D’Urso, P.; De Giovanni, L.; Massari, R.; D’Ecclesia, R. L.; Maharaj, E. A. Cepstral-Based Clustering of Financial Time Series. Expert Systems with Applications 2020, 161, 113705.

We get the comparison results as,

**Table 3 ISTA of different imaging series (K-means, K-medoids, Spectral, SOM)**

|  | *S/S_0_* | | | | *ln* (*S_t_/S_t-1_*) | | | |
| --- | --- | --- | --- | --- | --- | --- | --- | --- |
|  | K-me | K-mo | Spe | SOM | K-me | K-mo | Spe | SOM |
| Baseline ^[7]^ | **0.045** | - | - | **0** | - | - | - | - |
| RP | **0.015** | *0.001* | **0.008** | *0.001* | **0.013** | **0** | **0** | **0** |
| GRAF | 0.017 | **0** | 0.027 | 0.0004 | 0.019 | 0 | 0.018 | 0.0009 |
| GRDF | 0.021 | **0** | 0.021 | 0.0008 | 0.036 | 0.015 | 0.024 | 0.001 |
| MTF | 0.0131 | **0** | 0.019 | 0.0006 | 0.017 | 0.014 | 0.027 | 0.002 |

**Table 4 ISTA of different imaging series (K-means, K-medoids, Spectral, SOM)**

|  | *S/S_0_* | | | | *ln* (*S/S_t-1_*) | | | |
| --- | --- | --- | --- | --- | --- | --- | --- | --- |
|  | K-me | K-mo | Spe | SOM | K-me | K-mo | Spe | SOM |
| Baseline ^[7]^ | **0.045** | Null | Null | **0** | Null | Null | Null | Null |
| RP | **0.008** | 0.0002 | **0.019** | **0** | **0.0009** | **0** | **0** | **0.001** |
| GRAF | 0.013 | 0 | 0.027 | 0.0003 | 0.019 | 0 | 0.041 | 0.002 |
| GRDF | 0.009 | 0.002 | 0.021 | 0.0009 | 0.025 | 0 | 0.028 | 0.0006 |
| MTF | 0.010 | 0 | 0.026 | 0.0006 | 0.019 | 0 | 0.015 | 0.001 |
